# Calcium-binding protein immunoreactivity in Gudden’s tegmental nuclei and the hippocampal formation: differential co-localization in neurons projecting to the mammillary bodies

**DOI:** 10.3389/fnana.2015.00103

**Published:** 2015-08-04

**Authors:** Christopher M. Dillingham, Joshua D. Holmes, Nicholas F. Wright, Jonathan T. Erichsen, John P. Aggleton, Seralynne D. Vann

**Affiliations:** ^1^Behavioural Neuroscience, School of Psychology, Cardiff UniversityCardiff, UK; ^2^Visual Neuroscience and Molecular Biology, School of Optometry and Vision Sciences, Cardiff UniversityCardiff, UK

**Keywords:** calretinin, calbindin, diencephalon, dorsal tegmental nucleus, parvalbumin, postsubiculum, subiculum, ventral tegmental nucleus

## Abstract

The principal projections to the mammillary bodies arise from just two sites, Gudden’s tegmental nuclei (dorsal and ventral nuclei) and the hippocampal formation (subiculum and pre/postsubiculum). The present study sought to compare the neurochemical properties of these mammillary body inputs in the rat, with a focus on calcium-binding proteins. Neuronal calretinin (CR) immunoreactivity was sparse in Gudden’s tegmental nuclei and showed no co-localization with neurons projecting to the mammillary bodies. In contrast, many of the ventral tegmental nucleus of Gudden cell that project to the mammillary bodies were parvalbumin (PV)-positive whereas a smaller number of mammillary inputs stained for calbindin (CB). Only a few mammillary body projection cells in the dorsal tegmental nucleus of Gudden co-localized with PV and none co-localized with CB. A very different pattern was found in the hippocampal formation. Here, a large proportion of postsubiculum cells that project to the mammillary bodies co-localized with CR, but not CB or PV. While many neurons in the dorsal and ventral subiculum projected to the mammillary bodies, these cells did not co-localize with the immunofluorescence of any of the three tested proteins. These findings highlight marked differences between hippocampal and tegmental inputs to the rat mammillary bodies as well as differences between the medial and lateral mammillary systems. These findings also indicate some conserved neurochemical properties in Gudden’s tegmental nuclei across rodents and primates.

## Introduction

The mammillary bodies comprise one of a set of brain structures thought to be vital for human event memory (Dusoir et al., 1990; Vann and Aggleton, 2004; Tsivilis et al., 2008; Vann et al., 2009). Understanding their properties has, therefore, become necessary for the development of a comprehensive understanding of the neuroanatomy of memory. The principal inputs to the mammillary bodies in both rodents and primates come from just two sites: (1) the hippocampal formation (Nauta, 1956; Swanson and Cowan, 1977; Aggleton et al., 2005) and (2) Gudden’s tegmental nuclei (Hayakawa and Zyo, 1984; Allen and Hopkins, 1989; Saunders et al., 2012). Both sets of inputs are thought to contribute to learning and memory, but in fundamentally different ways (Vann, 2010). For this reason, the present study sought to compare the immunohistochemical properties of these direct inputs to the mammillary bodies. Attention focused on three different calcium-binding proteins, parvalbumin (PV), calbindin (CB), and calretinin (CR), which all bind Ca^2+^ with a high-affinity, acting as intracellular calcium buffers (Celio, 1990; Andressen et al., 1993). These calcium-binding proteins are expressed in specific subsets of neurons and often do not co-localize with one another, making them a useful tool for differentiating functional pathways (e.g., Rogers and Resibois, 1992; Gritti et al., 2003).

While the role of the hippocampal formation in learning and memory has received enormous attention, far less is known about that of Gudden’s tegmental nuclei. Gudden’s tegmental nuclei comprise two distinct divisions (Petrovicky, 1971): the dorsal tegmental nucleus (DTg) and the ventral tegmental nucleus (VTg). In the rat brain, their connections with the mammillary bodies are segregated so that DTg innervates the lateral mammillary nucleus, supporting navigation through its influence upon the head direction system (Vann and Aggleton, 2004; Vann, 2005, 2011; Taube, 2007; Clark et al., 2013; Dwyer et al., 2013), while VTg innervates the medial mammillary nucleus (Hayakawa and Zyo, 1984; Allen and Hopkins, 1989; Hopkins, 2005), again supporting spatial learning in the rat (Vann, 2009, 2013), but in ways that are different to the DTg pathway (Vann, 2010; Dillingham et al., 2015). It is known that some cells in both VTg and DTg of the rat are positive for markers of GABA, leu-enkephalin, and glutamate (Allen and Hopkins, 1989; Wirtshafter and Stratford, 1993; Gonzalo-Ruiz et al., 1999), which are also found on some neurons that innervate the mammillary bodies. The aim was to extend these connectional analyses to three calcium-binding proteins, PV, CB, and CR. A further reason to examine these calcium-binding proteins arises from recent evidence that PV immunoreactivity demarcates Gudden’s tegmental nuclei in the primate brain (Saunders et al., 2012).

The present study also sought to compare the tegmental inputs to the mammillary bodies with those from the hippocampal formation, given that both are implicated in learning, yet presumably make very different contributions. Analogous to the parallel mammillary body inputs that arise from VTg and DTg, hippocampal projections to the medial and lateral mammillary nuclei form parallel pathways. The medial mammillary nucleus is principally innervated by the dorsal and ventral subiculum (Allen and Hopkins, 1989). In contrast, the lateral mammillary nucleus primarily receives inputs from the postsubiculum (Allen and Hopkins, 1989; Yoder and Taube, 2011), which, like DTg, is a component of the head-direction system (Taube, 2007).

## Materials and Methods

### Subjects

The experiments described involved 12 male adult Lister Hooded rats weighing between 313 and 520 g (Harlan, UK) and four male adult Dark Agouti rats weighing from 216 to 245 g at the time of surgery (Harlan, UK; **Table 1**). Initially, mammillary body injections of the fluorescent retrograde tracers Fluorogold (Fluorochrome LLC, Denver, CO, USA) and Fast Blue (Polysciences, Eppelheim, Germany) were made in order to retrogradely label cells in Gudden’s tegmental nuclei that project to the mammillary bodies. Post-mortem, antibodies raised against the calcium-binding protein markers: PV (monoclonal, anti-mouse; Sigma–Aldrich, UK; supplier code – P3088), CR (monoclonal anti-mouse, Swant; supplier code: 6B3) and CB D28k (monoclonal anti-mouse, Swant; supplier code – 300) were used to determine (through fluorescent co-localization) the extent to which these projection neurons utilize these proteins.

**Table 1:** List of individual cases showing the strain of rat (LH, Lister Hooded; DA, Dark Agouti), the retrograde tracer injected into the mammillary bodies, the proportion of sections analyzed, the antibodies employed (CB, calbindin; CR, calretinin; PV, parvalbumin), and the regions of interest (ROI) that were scrutinized (DTg, dorsal tegmental nucleus of Gudden; Hpc, dorsal and ventral hippocampal formation; VTg, ventral tegmental nucleus of Gudden).

Case #	Strain	Tracer	Series	Antibody	ROI
75_11	LH	Fast Blue	1:5	CB, CR, PV	VTg/DTg/Hpc
75_12	LH	Fast Blue	1:5	CB, CR, PV	VTg/DTg/Hpc
157_15	LH	Fluorogold	1:3	PV	VTg/DTg
51_12	LH	Fluorogold	1:3	PV	VTg/DTg
52_20	DA	Fluorogold	1:3	CR	VTg/DTg
52_19	DA	Fluorogold	1:3	CB	VTg/DTg
52_34	DA	Fluorogold	1:3	CR, CB	VTg/DTg
53_3	DA	Fluorogold	1:3	CB	VTg/DTg
86_9	LH	Fast Blue	1:3	PV	VTg
86_1	LH	Fast Blue	1:3	PV	VTg
178_7	LH	Fast Blue	1:3	PV, CB	VTg/DTg/Hpc
178_2	LH	Fast Blue	1:3	PV, CR	VTg/DTg
74_9	LH	Fast Blue	1:3	CB	Hpc
74_10	LH	Fast Blue	1:3	PV	Hpc
75_5	LH	Fast Blue	1:3	PV	VTg/Hpc
75_6	LH	Fast Blue	1:3	PV, CR	VTg/Hpc

Animal husbandry and experimental procedures were conducted in accordance with the UK Animals (Scientific Procedures) Act, 1986 and associated guidelines, the EU directive 2010/63/EU, as well as the Cardiff University Biological Standards Committee.

### General Surgical Methods

Anesthesia was induced and maintained with isoflurane (4% and 1.5–2%, respectively; Sigma–Aldrich, Gillingham, UK)) combined with oxygen (2 L/minute). Animals were then placed in a stereotaxic frame (Kopf, Tujunga, CA, USA), with the mouth-bar set at +5.0 mm and, chloramphenicol eye ointment (Martindale Pharmaceuticals, Romford, UK) was topically applied to the eyes to protect the cornea. The analgesic Metacam (1 mg/kg; Boehringer Ingelheim, Germany) was administered subcutaneously before the scalp was incised and small openings were made in the skull and dura to allow access for a 0.5 μl Hamilton syringe (Hamilton, Bonaduz, Switzerland) containing Fluorogold or Fast Blue.

Tracer injections into the mammillary bodies were administered around the co-ordinates: anteroposterior –2.1, mediolateral ±0.8, and dorsoventral –10.4 from bregma, with slight variations made to encompass different subregions. Post surgery, animals received a 5 ml subcutaneous injection of 5% glucose in 0.9% saline (Baxter Healthcare Ltd, Norfolk, UK) and Dalacin antibiotic powder (Pharmacia Ltd., Kent, UK) was applied over the closed scalp incision. Animals were then allowed to recover in a thermostatically controlled chamber before being returned to individual housing with *ad libitum* access to food and water. General post-surgical health was monitored daily for the duration of the survival time.

### Fluorescent Tracer Injections

A total of 16 animals were injected with Fluorogold (*n* = 6) or Fast Blue (*n* = 10) into the mammillary bodies (**Table 1**). Fluorogold was made up as a 4% solution in distilled water while Fast Blue was made up as a 3% solution 0.1 M PBS. Following pressure injections of 0.04–0.05 μl into each site, the syringe was left in place for at least seven minutes in order to help limit tracer traveling back up the syringe tract.

### Immunohistochemistry

Following a postoperative period of 3–4 days, the animals were deeply anesthetized with sodium pentobarbital (Euthatal, Merial, Harlow, UK) and transcardially perfused with 0.1 M PBS (pH 7.4) at room temperature followed by 4% paraformaldehyde in 0.1 M PBS at ∼4°C. Brains were removed and post-fixed in the same solution for 4 h before being cryoprotected in a 25% solution of sucrose in 0.1 M PBS for 24 h prior to tissue sectioning. Brain tissue was kept in the dark at all times to prevent photobleaching of the tracer fluorescence. Brains were placed on a freezing platform and 40 μm coronal sections were cut on a sledge microtome (Leica 1400). A 1-in-3 or 1-in-5 series of sections from each brain was mounted directly onto gelatin-subbed slides, and allowed to dry in the dark at room temperature. The first series was stained with cresyl violet to allow for both localization of injection sites and comparative architectural measures of Gudden’s nuclei with fluorescent sections. The remaining series were either reacted immediately with antibodies raised against PV, CB, and CR, or stored in cryoprotectant at –20°C prior to immunohistochemistry.

Tissue was washed in 0.1 M PBS (pH 7.4) to remove cryoprotectant (if necessary) before being treated with a blocking buffer containing 3–5% normal horse serum (S-2000, Vector Laboratories, UK) in 0.1 M PBS and agitated on a stirrer for between 30 min and 2 h. Sections were subsequently incubated in primary antibody solution (1:10,000 dilutions in 0.2% Triton-X-100 in PBS containing 1% normal horse serum), for 24 h at room temperature. The tissue underwent further washes in 0.1 M PBS, and to complete the reaction, the tissue was incubated in a secondary antibody solution (Dylight-594; horse, anti-mouse; 1:200 dilution in 0.2% Triton-X-100 in 0.1 M PBS containing 1% normal horse serum) overnight on a shaker table at room temperature. Following an additional series of washes in 0.1 PBS, the tissue sections were mounted on gelatin-subbed slides, allowed to dry for 1–2 days in the dark, and coverslipped using DPX mounting medium (Raymond A Lamb, UK). A Leica DM6000 B microscope was used for fluorescence microscopy. An attached Leica DFC350 FX digital camera with acquisition software (LAS AF image, Leica) was used to capture images. Control sections were treated with an identical procedure to those above, but in the absence of the primary antibody. Non-specific staining was not observed. All image analysis was performed in Fiji (‘*Fiji is just imageJ*’; freely accessible software available from http://fiji.sc/Fiji). The method for identification of cells co-localizing tracer and immunofluorescence was multifaceted. Initially, double-labeling was determined through the identification of overlapping signal, e.g., white signal resulting from blue and yellow pseudo-color apportioned to independent cubes. Subsequently, each cell was identified in unmerged channels in order to distinguish between overlapping and true co-localization of fluorescence. Finally, if these approaches were not conclusive for a given cell, single line gray scale saturation profiles of tracer and immunofluorescence were overlaid and compared in terms of amplitude and width of peaks relative to background levels.

### Anatomical Nomenclature and Borders

Anatomical names and borders follow the descriptions of Gudden’s tegmental nuclei by Hayakawa and Zyo (1983). Consequently, the DTg is divided into a pars ventralis [corresponding to the pars centralis of Petrovicky (1971)] and a pars dorsalis [corresponding to the pars pericentralis of Petrovicky (1971)]. The VTg is predominantly composed of the pars principalis (Hayakawa and Zyo, 1983). The terminology for the retrosplenial cortex follows Van Groen and Wyss (2003). For other structures, the terminology follows Swanson (1992). One example concerns the borders of the subiculum, presubiculum, parasubiculum, and postsubiculum. The laminae descriptions for the subiculum match those of Kloosterman et al. (2003), so that the subiculum consists of a superficial molecular layer and a deeper, thick layer of pyramidal cells. The term postsubiculum is used (Van Groen and Wyss, 1990), while recognizing that some authorities regard this region as part of the presubiculum (e.g., Witter, 2002). For this reason, the postsubiculum is regarded as having six layers (Van Groen and Wyss, 1990), including a cell sparse lamina dissecans (layer IV).

## Results

### Dorsal Tegmental Nucleus of Gudden (DTg) – Calcium-Binding Proteins

**FIGURE 1:**
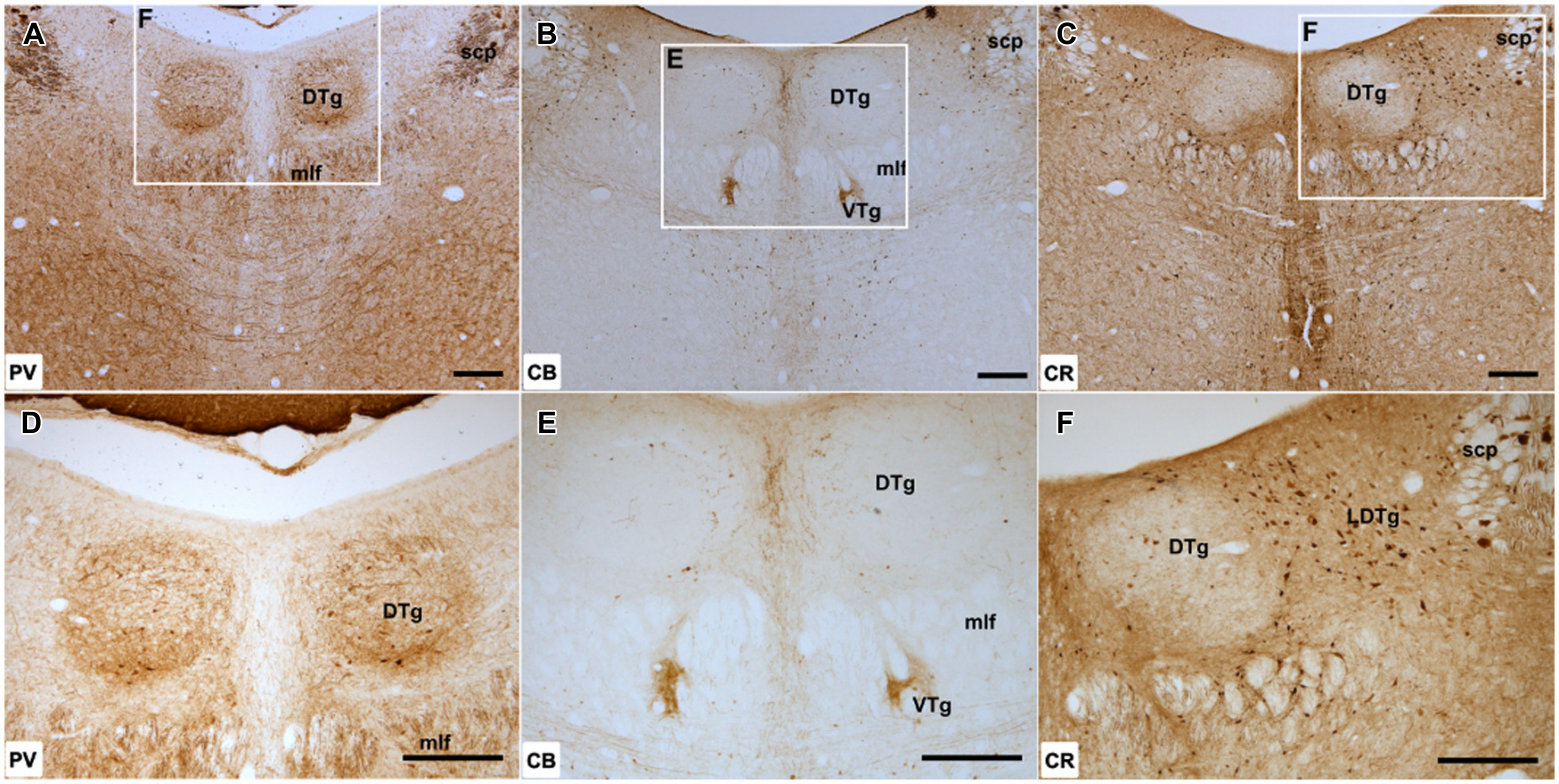
**Dorsal tegmental nucleus of Gudden: **(A–C)**, Low magnification (50× magnification) photomicrograph of parvalbumin (PV; **A**), calbindin (CB; **B**), and calretinin (CR; **C**) immunoreactivity in coronal sections of the rat midbrain.** PV label was present throughout DTg, but the densest cellular and neuropillar label was confined to the pars ventralis division of the nucleus. CB immunoreactivity was virtually absent within the dorsal tegmental nucleus, while only sparsely distributed CR immunoreactive cells were found. **(D–F)**, Higher magnification (100× magnification) photomicrographs of regions corresponding to those denoted by white rectangles in **(A–C)**. Abbreviations: DTg, dorsal tegmental nucleus of Gudden; LDTg, laterodorsal tegmental nucleus; mlf, medial longitudinal fasciculus; scp, superior cerebellar peduncle; VTg, ventral tegmental nucleus of Gudden. Scale bars: 250 μm.

Both cellular and neuropillar immunoreactivity to PV were present within DTg, clearly defining the boundary of the nucleus from neighboring tegmental and raphe nuclei. The density of immunoreactivity was non-uniform, with the strongest signal confined predominantly within the pars ventralis subdivision. In the dorsal part of DTg, PV labeled neurons were evident but were more sparsely distributed, while neuropillar label was comparatively weaker (**Figures 1A,D**).

Calbindin immunoreactivity was not present within DTg with the exception of a few medially located CB-positive cells. The distribution of these few labeled cells appeared to extend laterally from the midline CB-positive neurons of the dorsal raphe nuclei (**Figures 1B,E**).

Calretinin immunoreactive cells were present, but only in small numbers and appeared to be confined to the ventromedial aspect of the nucleus. Given their proximity, these cells were potentially ectopic neurons of the neighboring CR immunoreactive dorsal raphe nuclei situated within the boundary of DTg. Dense CR immunoreactivity was also present within the neighboring laterodorsal tegmental nucleus (**Figures 1C,F**). As a consequence, DTg stood out because of its lack of CR staining.

### Ventral Tegmental Nucleus of Gudden (VTg) – Calcium-Binding Proteins

**FIGURE 2:**
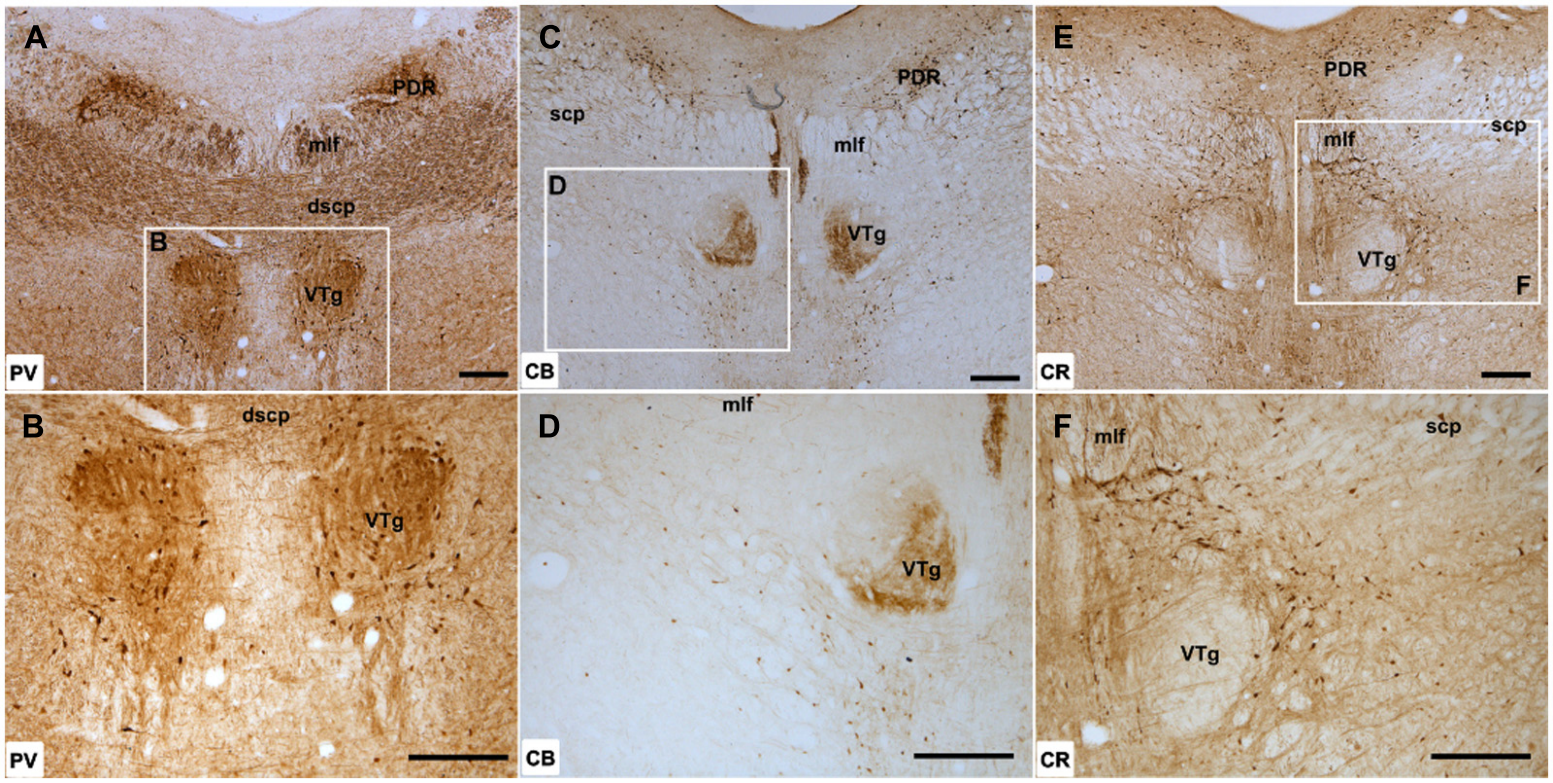
**Ventral tegmental nucleus of Gudden: **(A,B)** Low magnification (**A**; 50× magnification) and higher magnification (**B**; 100× magnification) photomicrographs of PV immunoreactivity in coronal sections of the rat midbrain.** Dense PV label was present in the rostral part of the VTg but further caudally, an apparent boundary of neuropil immunoreactivity was present with more labels present in the lateral half of the nucleus. **(C,D)** Low (**C**; 50×) and high magnification (**D**; 100×) photomicrographs of CB immunoreactivity, showing dense, selective neuropil label in the ventromedial part of VTg but the absence of label in the dorsolateral portion of the nucleus. **(E,F)** Low (**E**; 50×) and high magnification (**F**; 100×) photomicrographs of CR immunoreactivity, showing the virtual absence of neuronal or neuropillar immunoreactivity throughout VTg. Abbreviations: dscp, decussation of the superior cerebellar peduncle; mlf, medial longitudinal fasciculus; PDR, posterodorsal raphe nucleus; scp, superior cerebellar peduncle. Scale bars: 250 μm.

Parvalbumin immunoreactivity was present throughout VTg, with dense neuropil label throughout the rostral part of the nucleus and an appreciable number of PV immunoreactive cells (**Figures 2A,B**). In the caudal part of VTg, the density of neuropil and labeled cells present was greater laterally than medially.

Neuropillar CB immunoreactivity was present throughout the rostral part of VTg. Further caudally, a distinct boundary could be observed between ventromedial and dorsolateral parts of the nucleus, with the former positive for neuropillar CB immunoreactivity and the latter negative (**Figures 2C,D**). This border did not appear to match any cytoarchitectonic divisions within VTg. There was a moderate amount of cell body label across VTg at both rostral and caudal levels.

Calretinin immunoreactivity was generally weak within the ventral tegmental nuclei, with few CR-positive cells present (**Figures 2E,F**). Neuropil label appeared stronger in the rostral part of the nucleus, while in the caudal VTg, both neuropil and cellular immunoreactivity for CR were absent (**Figures 2E,F**).

### Hippocampal Formation – Calcium-Binding Proteins

**FIGURE 3:**
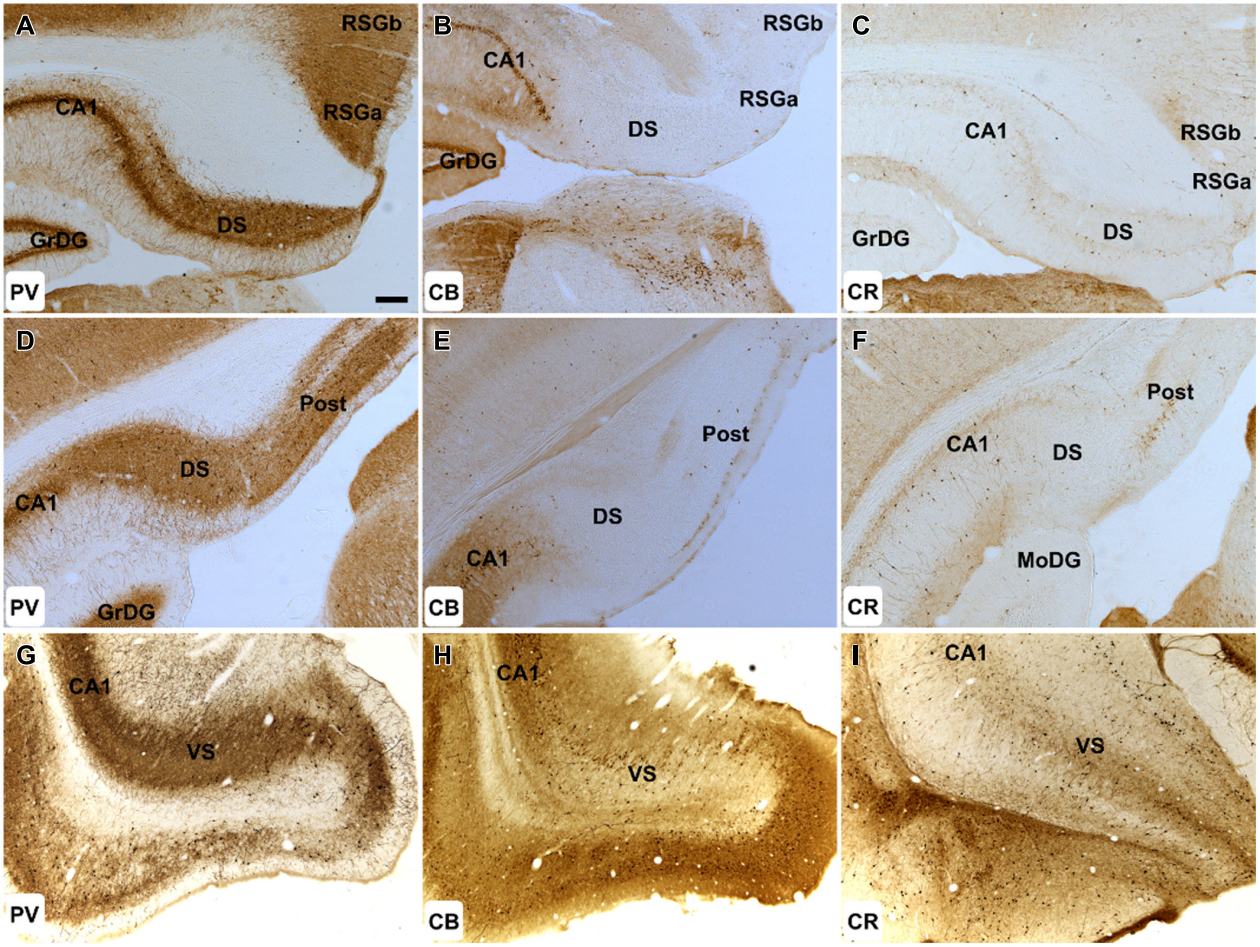
**Hippocampal formation: **(A–C)** 50× magnification photomicrographs of PV **(A,D,G)**, CB **(B,E,H)**, and CR **(C,F,I)** immunoreactivity in coronal sections of the rat cerebrum.** Cellular and neuropillar PV label was present throughout the DS, VS, and post. CB immunoreactivity was virtually absent within these structures. While only sparsely distributed CR immunoreactive cells were found in the DS, the postsubiculum exhibited strong cellular immunoreactivity. Abbreviations: CA1, field CA1 of the hippocampus; DS, dorsal subiculum; GrDG, granular layer of the dentate gyrus; MoDG, molecular layer of the dentate gyrus; Post, postsubiculum; RSGa, retrosplenial granular cortex, a region; RSGb, retrosplenial granular cortex, b region; VS, ventral subiculum. Scale bar (applies to all): 200 μm.

Both dense neuropillar and cell body PV immunoreactivity were present throughout the dorsal subiculum and postsubiculum (**Figures 3A,D**). In the ventral subiculum, neuropil immunoreactivity was dense, however, the distribution of PV immunoreactive cells was low, particularly proximal to the CA1 border (**Figure 3G**).

Calbindin immunoreactivity was virtually absent in both the proximal and distal subiculum. Cellular and neuropillar signal, albeit sparse, was greatest proximal to the border of the subiculum with CA1 (<6 cells/section in each hemisphere; **Figures 3B,E**). CB positive cells were present in layer III of the postsubiculum but were infrequent (10–20/section; **Figure 3E**), while in layer II, dense neuropil and cell body label was observed (**Figure 3E**). In the ventral subiculum, the distribution of neuronal CB label was densest in the superficial part of the pyramidal layer whereas neuropil immunoreactivity was absent in the deep pyramidal layer but dense in the molecular and superficial pyramidal layers (**Figure 3H**).

Sparsely distributed CR immunoreactive cells were present in the pyramidal layer of the dorsal and ventral subiculum while CR neuropil immunoreactivity was confined to the deepest parts of the pyramidal layer and was densest proximal to the border of the CA1 (**Figures 3C,I**). In the postsubiculum, a dense localized patch of cellular and neuropillar CR immunoreactivity was observed spanning layer III, while sparse neuropillar immunoreactivity was present in layers V–VI. In both cases, label was densest on the distal dorsal subiculum/postsubiculum boundary (**Figure 3F**).

### Mammillary Body Inputs – Gudden’s Tegmental Nuclei/Hippocampal Formation

**FIGURE 4:**
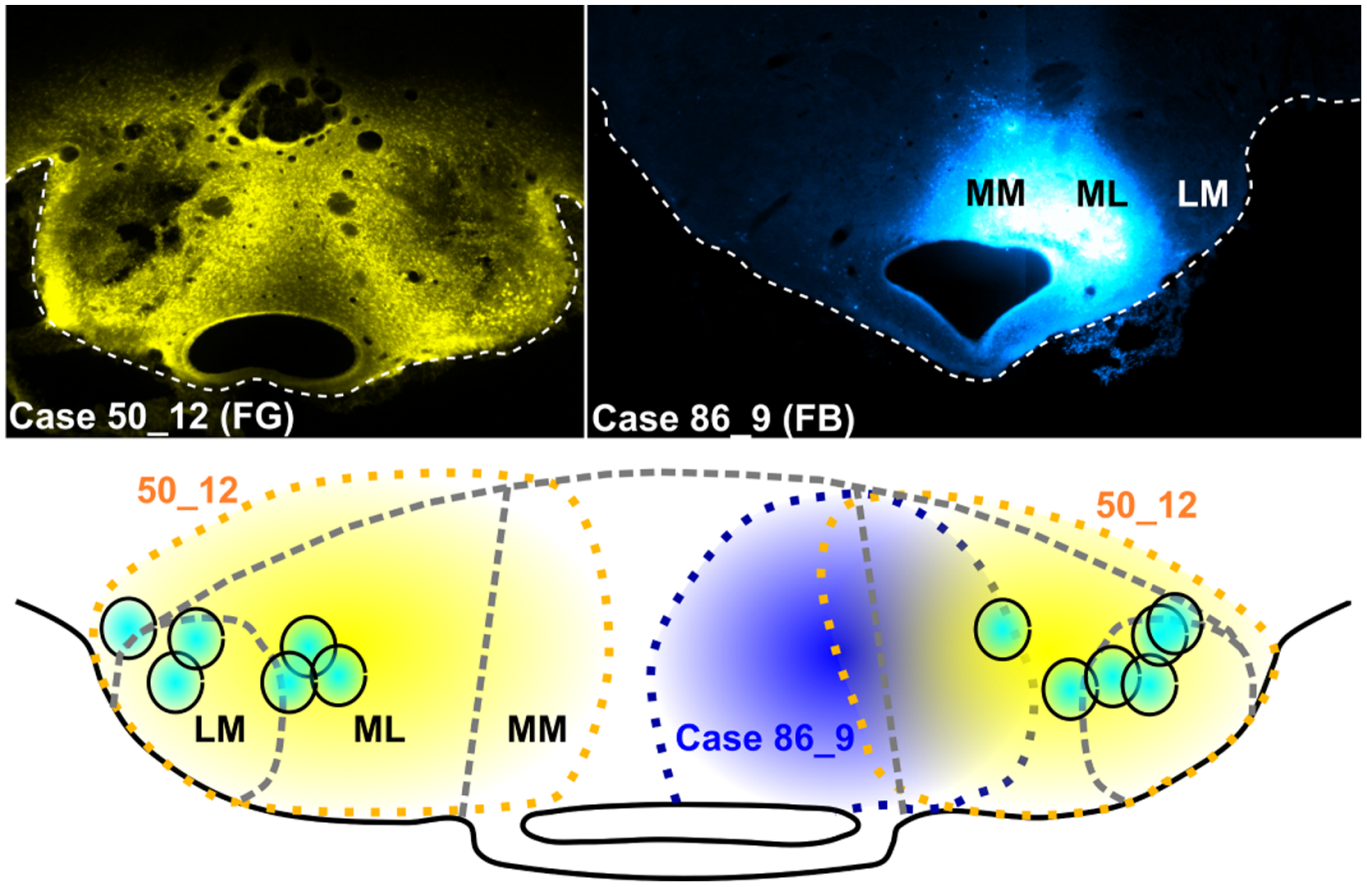
**Mammillary body injection sites: Photomicrographs of two representative cases, one with bilateral Fluorogold (FG) injections (**upper left**; case 50_12) and the other, a unilateral injection of Fast Blue (FB) localized to the right medial mammillary body nucleus (**upper right**; case 86_9).** The schematic diagram shows the spread of tracer in case 51_12 (yellow dashed lines and fill) and case 86_9 (blue dashed lines and fill), while turquoise circles show the mammillary body injection sites for a representative selection of six cases (all bilateral injections).

Retrograde tracer injections in the mammillary bodies were typically centered in either the medial mammillary nucleus (pars lateralis) or the lateral mammillary nucleus, though nearly always extended across both nuclei (**Figure 4**). This spread ensured that the Fluorogold/Fast Blue injections usually reached both pars medialis of the medial mammillary nucleus and the lateral mammillary nucleus. In two cases (86_1 and 86_9), however, a unilateral injection was confined to pars medialis of the medial mammillary nucleus (**Figure 4**).

**FIGURE 5:**
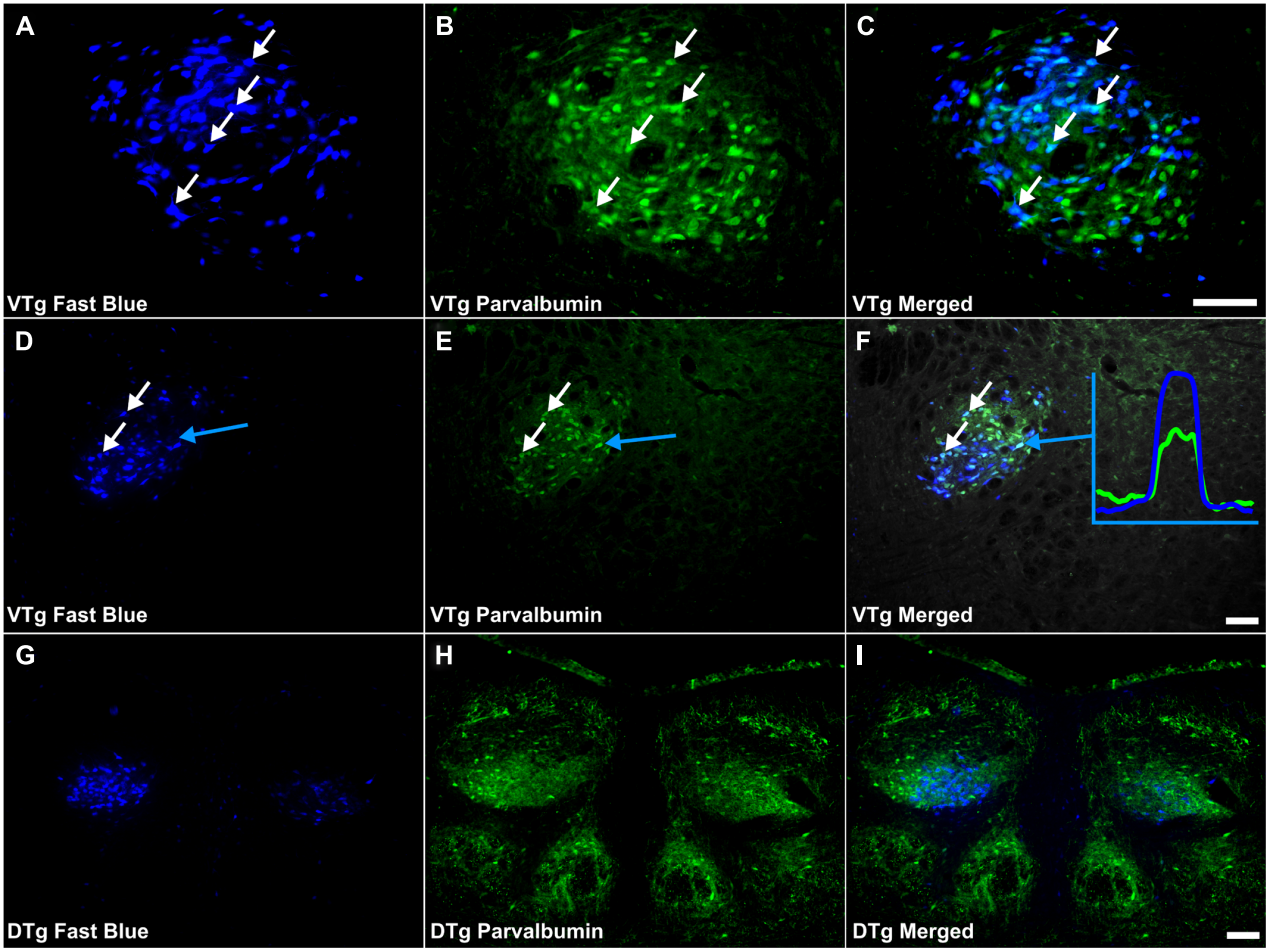
**Parvalbumin/Gudden’s tegmental nuclei: **(A,D,G)** Fluorescent photomicrographs showing retrogradely labeled Fast Blue cells in the rostral VTg, more caudal **(D)** parts of VTg and in the DTg **(G)**, respectively. (B,E,F)** show fluorescent PV immunoreactivity in respective sections while **(C,F,I)** show retrograde Fast Blue label and PV immunoreactivity superimposed. White arrows in **(A–C)** and **(D–E)** identify examples of individual retrogradely Fast Blue labeled cells **(A,D)** that co-localized PV **(B,F)** and the resulting co-localization of overlapping signal **(C,F)**. Further confirmation of colocalization came from visualization of the overlapping grayscale peaks of tracer (blue) and PV (green), an example of which is shown in **(F).** A considerable proportion of those neurons in VTg, projecting to the mammillary bodies, i.e., retrogradely labeled cells, were found to co-localize PV, however, far fewer equivalent cells were observed in DTg. Scale bars: 100 μm.

Bilateral injections of Fluorogold or Fast Blue into the mammillary bodies resulted in substantial retrograde labeling of both the VTg and DTg (e.g., **Figures 5A,G**, respectively). Caudally through the midbrain tegmentum, retrogradely labeled cells were first encountered within VTg, loosely distributed within the fibers of the medial longitudinal fasciculus. The caudal apices of the VTg extended dorsally to reach the rostral boundary of the DTg. The rostral-most retrogradely labeled cells within DTg were localized within pars dorsalis, but further caudally within the nucleus, retrograde cell body label was confined to the pars ventralis. The presence of retrogradely labeled cells in both DTg and VTg is consistent with the injections involving both the lateral and medial mammillary nuclei.

In the subicular cortices, tracer injections into the mammillary bodies, in all cases, resulted in densely packed retrogradely labeled neurons. In those cases in which the injections were centered more laterally in the lateral mammillary body nuclei, retrograde label was particularly dense in more distal regions of the dorsal subiculum and in the adjacent postsubiculum. In those cases with more medial injections, i.e., cases 86_1 and 86_9, retrogradely labeled cells were most densely distributed in proximal regions of the dorsal subiculum and absent in the postsubiculum. Comparably, in the ventral subiculum, medial mammillary body injections resulted in distributions of retrograde cell body label that were densest proximal to CA1, while larger, less specific injections resulted in less proximal-distal specificity.

### Double-Labeling in Gudden’s Tegmental Nuclei

**FIGURE 6:**
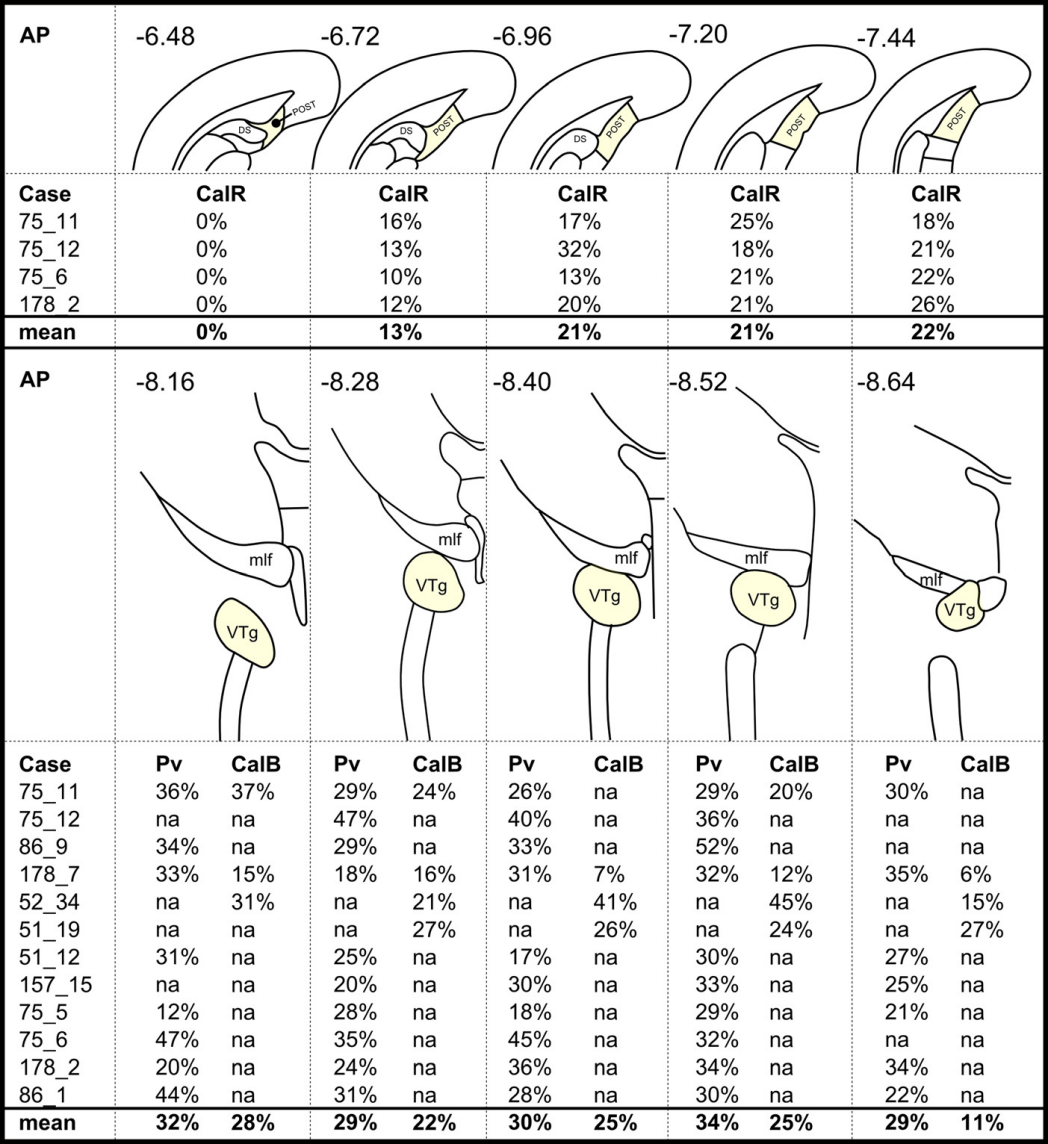
**A case-by-case summary showing the percentage of retrogradely labeled neurons that colocalized: **(Top)** calretinin (CalR) in the postsubiculum (POST) and **(Bottom)** parvalbumin (Pv)/calbindin (CalB) in the VTg.** Percentage values in each column are averages across two hemispheres of a given section and relate to different rostral-caudal locations as shown by the corresponding anatomical schematic diagrams and the rostral-caudal brain atlas coordinates (AP; Paxinos and Watson, 2005). Missing values (‘na’ in this Figure) show when corresponding sections were not available in a series or if a given case had not been reacted for the protein in question. Abbreviations: DS, dorsal subiculum; mlf, medial longitudinal fasciculus.

Co-localization of PV with retrogradely labeled neurons within Gudden’s tegmental nuclei was evident in all nine reacted cases (**Figures 5A–I**). In each case, numerous double-labeled neurons were observed in the VTg, with no obvious topography along the rostral-caudal axis (**Figure 6**). Within DTg, only ∼1% of retrogradely labeled cells co-localized PV (**Figures 5G–I**). In the two cases in which retrograde cell body label was absent in DTg, i.e., cases 86_1 and 86_9, only pars medialis of the medial mammillary nucleus was injected (**Figure 6**). Similarly, PV immunoreactivity was also present, exclusive of fluorescent retrograde label, in neurons of both VTg and DTg (**Figure 5C**).

**FIGURE 7:**
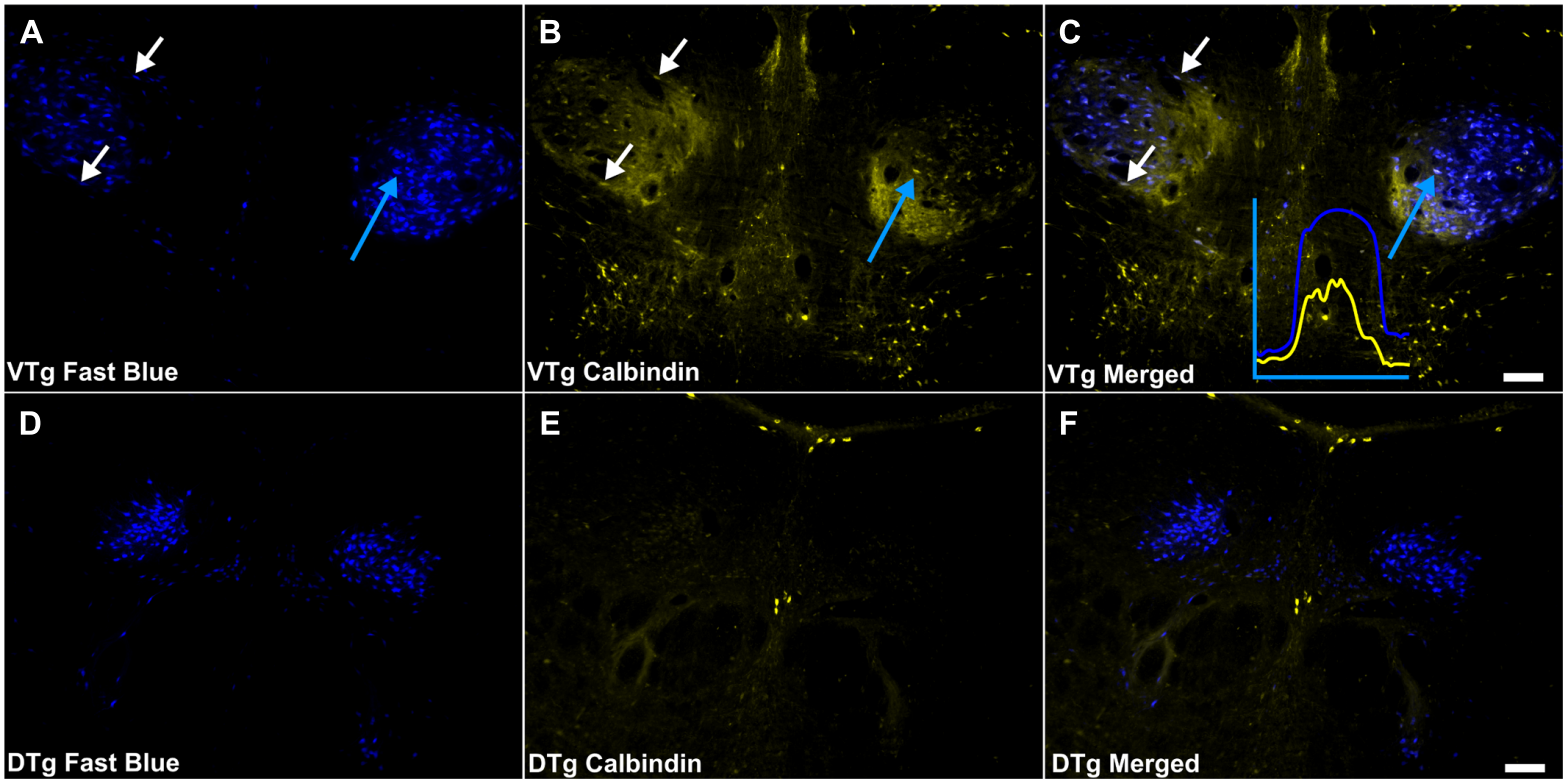
**Calbindin/Gudden’s tegmental nuclei: **(A,D)** Fluorescent photomicrographs showing retrograde Fast Blue cell body label in the VTg and dorsal tegmental nuclei of Gudden (DTg), respectively. (B,E)** show fluorescent CB immunoreactivity in respective sections while **(C,F)** show retrograde Fast Blue label and CB immunoreactivity superimposed. White arrows in **(A–C)** identify examples of individual retrogradely Fast Blue labeled cells **(A)** that co-localized CB **(B)** and the resulting co-localization of signal **(C)**. Further confirmation of colocalization came from visualization of the overlapping grayscale peaks of tracer (blue) and CB (yellow), an example of which is shown in **(C).** Moderate neuropillar CB immunoreactivity was present in VTg and was densest ventromedially. Neuronal CB immunoreactivity was present and a notable number of these cells was found to co-localize retrogradely labeled Fast Blue fluorescence. In DTg, CB immunoreactivity was virtually absent except for a few centrally located neurons, which did not co-localize retrogradely transported Fast Blue fluorescence. Scale bars: 100 μm.

Calbindin immunoreactivity was all but absent within the boundaries of DTg, with the exception of a few centrally located neurons, which did not co-localize with retrogradely transported Fluorogold or Fast Blue fluorescence (**Figures 7D–F**). In VTg (**Figure 6**), dense neuropillar CB immunofluorescence was only present within the ventromedial portion of VTg, while a moderately dense distribution of neuronal label was present throughout the nucleus (**Figures 2C,D**). The latter resulted in overlying distributions of the CB and Fluorogold/Fast Blue signals, with a considerable proportion co-localizing the two fluorescent signals (**Figures 6** and **7A–C**).

**FIGURE 8:**
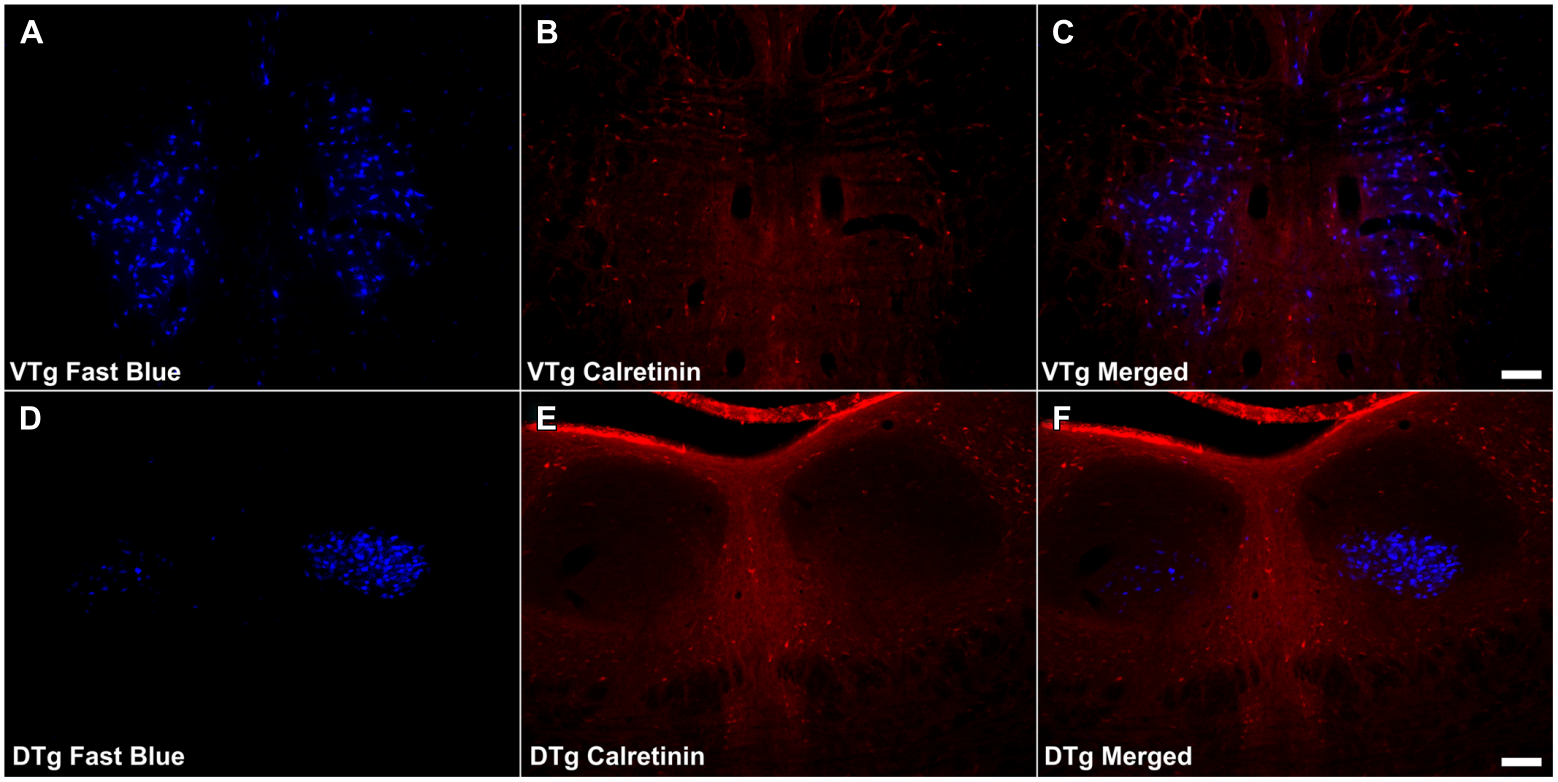
**Calretinin/Gudden’s tegmental nuclei: **(A,D)** Fluorescent photomicrographs showing retrograde Fast Blue cell body label in the VTg and DTg, respectively. (B,E)** show fluorescent CR immunoreactivity in respective sections while **(C,F)** show retrograde Fast Blue label and CR immunoreactivity superimposed. CR neuronal immunoreactivity was found to be sparse in VTg, and as a result, there was minimal co-localization with retrograde Fast Blue label. In DTg, CR neuronal immunoreactivity was all but absent, and again, no co-localization was observed. Scale bars: 100 μm.

Calretinin immunoreactivity was present within the boundaries of VTg, often in close proximity to or overlapping with the retrogradely labeled Fluorogold/Fast Blue neurons. Even so, very infrequent co-localization of the two cell populations was observed (**Figures 8A–C**). CR immunoreactive neuropil and cells were virtually absent in DTg, and no co-localization of CR and Fluorogold/Fast Blue signal was observed (**Figures 8D–F**).

### Double-Labeling in the Hippocampal Formation

**FIGURE 9:**
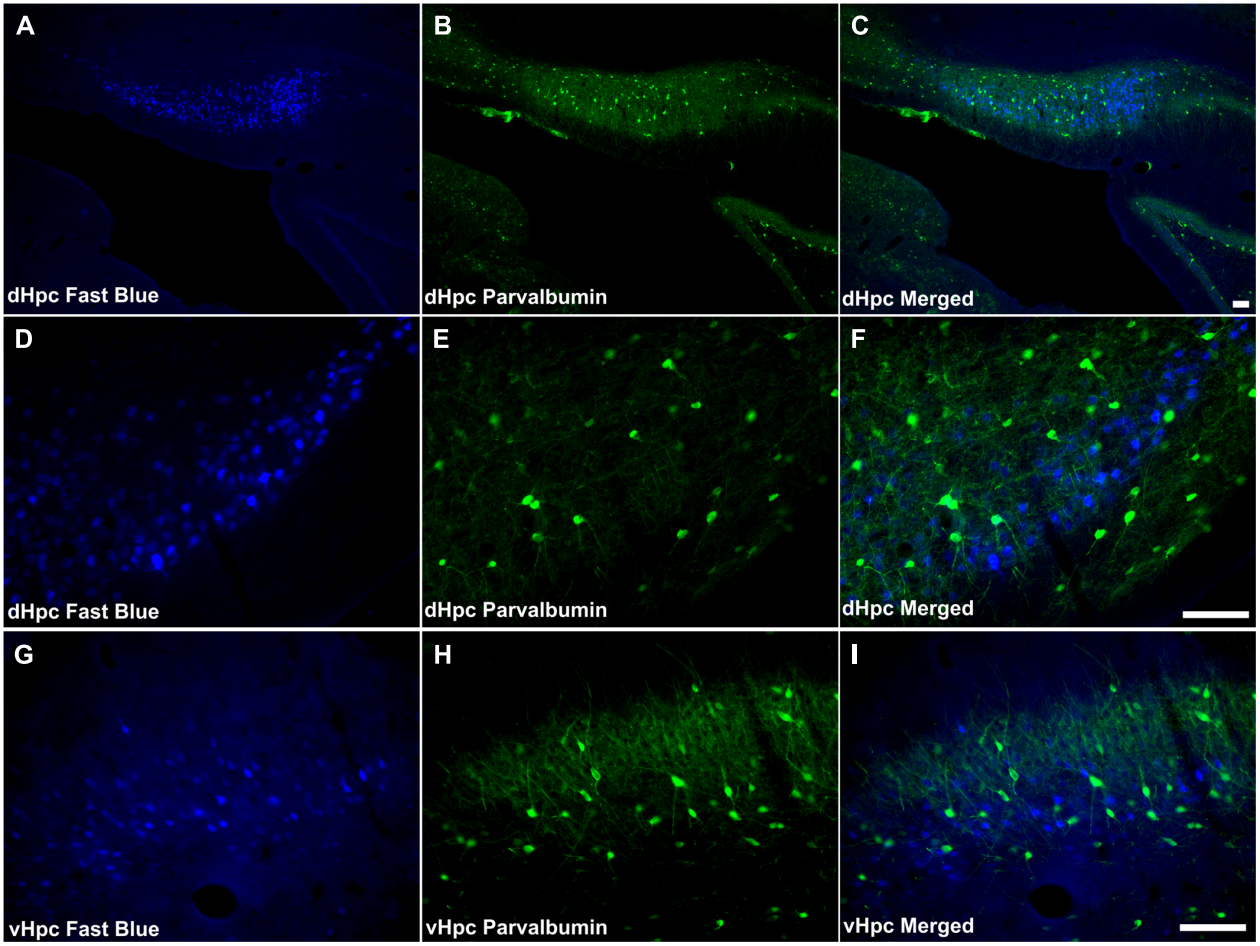
**Parvalbumin/hippocampal formation: **(A,D,G)** Fluorescent photomicrographs showing retrograde Fast Blue cell body label in the DS, Post, and VS, respectively. (B,E,H)** show fluorescent PV immunoreactivity in respective sections while **(C,F,I)** show retrograde Fast Blue label and PV immunoreactivity superimposed. Dense neuropillar and neuronal PV immunoreactivity were present in the subicular cortices, however there was virtually no co-localization with retrogradely labeled Fast Blue neurons was apparent. Abbreviations: dHPC, dorsal hippocampal formation; vHpc, ventral hippocampal formation. Scale bars: 100 μm.

A very small number of PV immunoreactive neurons were found to co-localize retrogradely transported Fast Blue in the dorsal subiculum (**Figures 9A–F**). These cells (<2 cells/case) were confined to the proximal subiculum. Although the distribution of neuronal label was dense, no double-labeled neurons were found in the distal subiculum or in the postsubiculum. Similarly, in the ventral subiculum, although a considerable number of neurons were PV immunoreactive, no co-localization of immunofluorescence with retrograde fluorescence was observed (**Figures 9G–I**).

**FIGURE 10:**
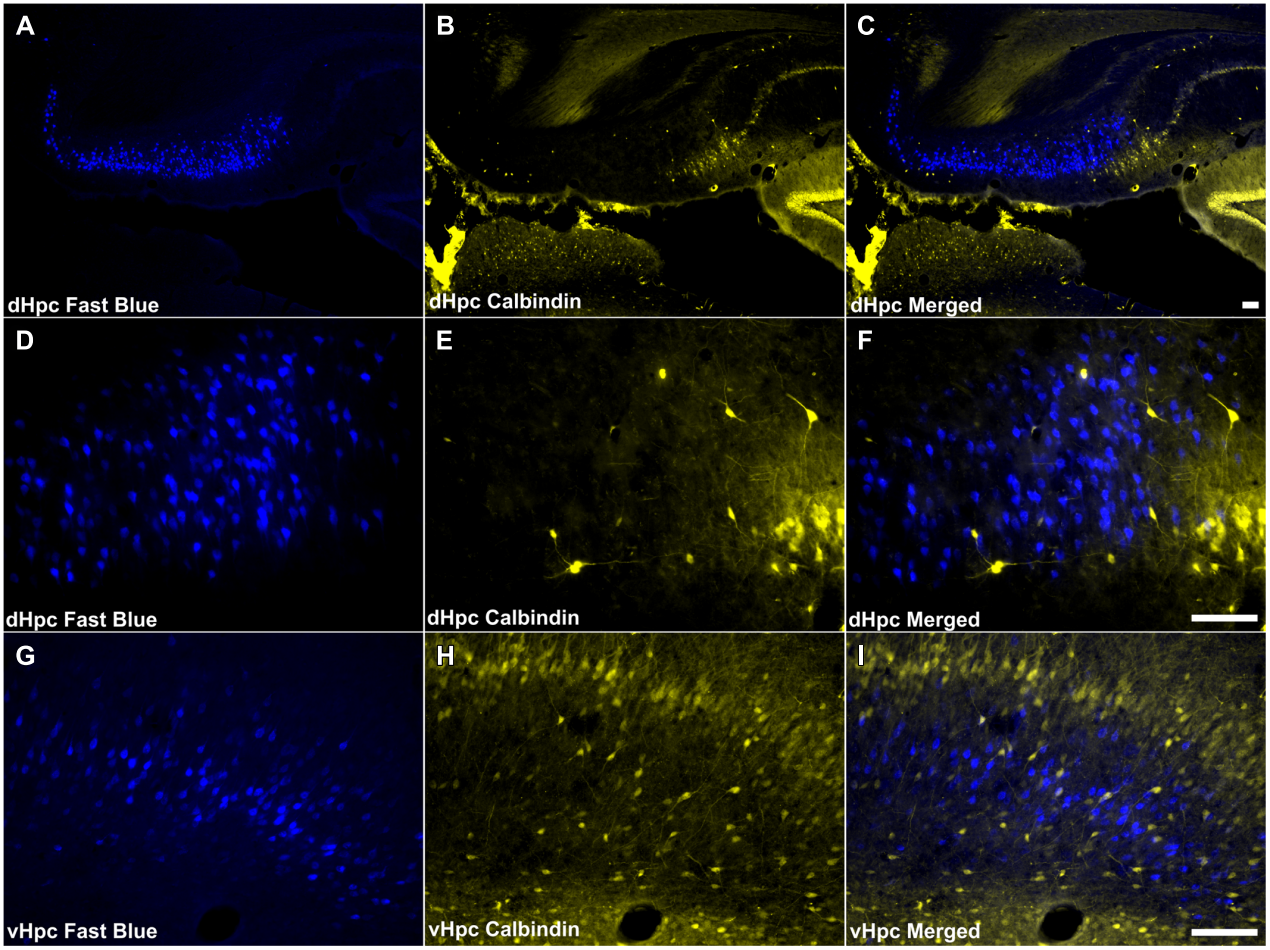
**Calbindin/hippocampal formation: **(A,D,G)** Fluorescent photomicrographs showing retrograde Fast Blue cell body label in the dorsal subiculum at low (50×) and high (200×) magnification, as well as the ventral subiculum (200×). (B,E,H)** show fluorescent CB immunoreactivity in respective sections while **(C,F,I)** show retrograde Fast Blue label and CB immunoreactivity superimposed. CB immunoreactive cells in the subicular cortices were infrequent and sparsely distributed, and no co-localization of retrograde fluorescence and immunofluorescence was observed. Abbreviations: dHPC, dorsal hippocampal formation; vHpc, ventral hippocampal formation. Scale bars: 100 μm.

**FIGURE 11:**
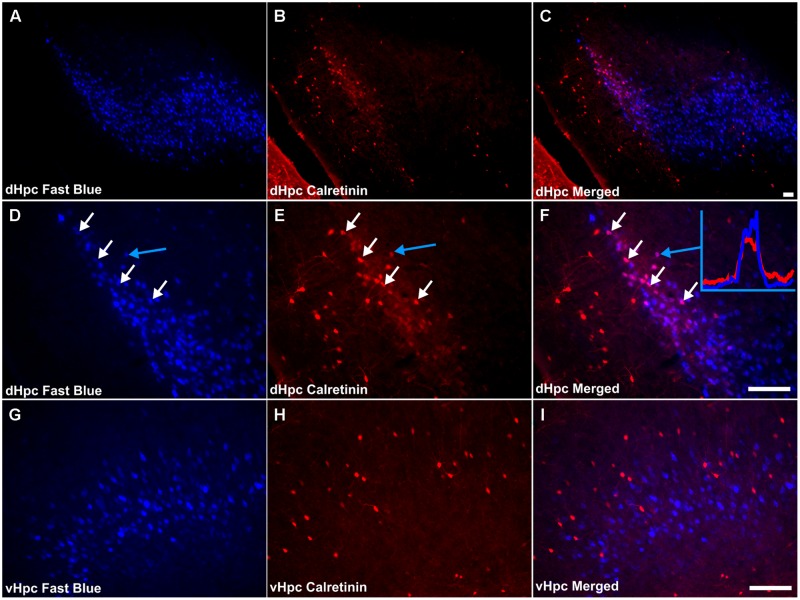
**Calretinin/hippocampal formation: **(A,D,G)** Fluorescent photomicrographs showing retrograde Fast Blue cell body label in the subicular cortices (dorsal subiculum and postsubiculum at low (50×) and high (200×) magnification, as well as the ventral subiculum (200×). (B,E,H)** show fluorescent CR immunoreactivity in respective sections while **(C,F,I)** show retrograde Fast Blue label and CR immunoreactivity superimposed. Although largely sparse, or absent throughout the subicular cortices, a dense localized region of CR immunoreactivity was present in layer III of the postsubiculum, proximal to the dorsal subiculum, in which a high proportion of co-localization with retrogradely labeled fast blue cells was encountered. White arrows in **(D–F)** identify examples of individual retrogradely Fast Blue labeled cells **(D)** that co-localized CR **(E)** and the resulting co-localization of signal **(F)**. Further confirmation of colocalization came from visualization of the overlapping grayscale peaks of tracer (blue) and CR (red), an example of which is shown in **(F)**. Abbreviations: dHPC, dorsal hippocampal formation; vHpc, ventral hippocampal formation. Scale bars: 100 μm.

Consistent with the virtual absence of CB immunoreactivity in the dorsal subiculum and postsubiculum, no double-labeled cells were observed (**Figures 10A–F**). In the ventral subiculum, while the number of CB immunoreactive neurons was considerably higher, again, no co-localization with retrograde immunofluorescence was observed (**Figures 10G–I**). Similarly, neither the CR immunoreactivity in the dorsal subiculum, limited as it was (**Figures 11A–F**), nor the greater density of CR cells in the ventral subiculum (**Figures 11G–I**) were found to overlap with the distribution of retrogradely labeled Fast Blue neurons. As a result, no co-localization with retrograde fluorescence was observed in either neuronal population (**Figures 11A–I**). In contrast, an appreciable proportion of the CR immunoreactive cells in layer III of the postsubiculum were found to co-localize with Fast Blue label (**Figures 11D–F**). Again, co-localization of immunofluorescence showed no topography along the rostral-caudal axis (**Figure 6**) and was present at consistent levels throughout layer III of the postsubiculum with the exception of its rostral-most apex, where it was absent (**Figure 6**).

## Discussion

There has been a resurgence of interest in the mammillary bodies in response to recent studies that have highlighted their importance for human episodic memory (Carlesimo et al., 2007; Tsivilis et al., 2008; Vann et al., 2009; Rosenbaum et al., 2014). In order to understand better their roles in learning and memory, more knowledge is required concerning the properties of the connections of these hypothalamic nuclei. The mammillary bodies have two major afferent sources: the hippocampal formation and Gudden’s tegmental nuclei (Nauta, 1956; Swanson and Cowan, 1977; Hayakawa and Zyo, 1984; Allen and Hopkins, 1989; Hopkins, 2005; Saunders et al., 2012). In both the rat and macaque monkey, dense hippocampal projections to the mammillary bodies arise from the subicular complex (e.g., Swanson and Cowan, 1977; Aggleton et al., 2005; Wright et al., 2010) while tegmental projections arise from the dorsal and ventral nuclei of Gudden (e.g., Hayakawa and Zyo, 1984; Allen and Hopkins, 1989; Saunders et al., 2012). Of these projections, only the tegmental connections with the mammillary bodies are reciprocal. In the rat brain, it is known that the DTg is interconnected with the lateral mammillary nucleus while the VTg is interconnected with the medial mammillary nucleus (Hayakawa and Zyo, 1984; Allen and Hopkins, 1989; Hopkins, 2005). The present study sought to compare these two major inputs (tegmental and hippocampal) to the mammillary bodies by combining fluorescent immunohistochemistry for three calcium-binding proteins (PV, CB, and CR) with fluorescent retrograde pathway tracing.

### Gudden’s Tegmental Nuclei

The three calcium-binding proteins under investigation had very different patterns of expression across Gudden’s tegmental nuclei. CR immunoreactivity was either absent or very light in both DTg and VTg. Moreover, while there was minimal CB immunoreactivity in DTg, there was restricted dense neuropil label in VTg and also some staining of cell bodies. Of the three markers, PV was the most prominent in both DTg and VTg with dense neuropil immunoreactivity, as well as neuronal label, in both regions. There was some evidence that the neuropil within VTg showed a complementary pattern of staining across calcium-binding proteins, with CB most dense in the ventromedial portion and PV densest in the dorsolateral portion, a distinction that does not appear to match a known cytoarchitectonic division within the nucleus (Petrovicky, 1971). In addition, the overall pattern of PV staining bears a strong resemblance to that reported in the cynomolgus monkey (*Macaca fascicularis*) in which PV immunoreactive neurons were found in both the DTg and VTg (Saunders et al., 2012). A further similarity was found with CR expression, which was absent in both rat and macaque tegmental nuclei of Gudden. There was, however, a discrepancy in CB staining across species; while present in rat VTg, this marker was absent across both VTg and DTg in the macaque (Saunders et al., 2012).

In the macaque monkey study, due to methodological constraints, it was not possible to determine whether individual PV-positive cells comprise the *same* population of cells that innervate the mammillary bodies (Saunders et al., 2012). However, the present study addressed this issue by combining fluorescent retrograde pathway tracing and immunofluorescence. In rat DTg and VTg, there are neurons that both project to the mammillary bodies and also display immunoreactivity for PV, i.e., co-localization of immunofluorescent and retrograde fluorescent signal. It may, therefore, be the case that these PV-positive cells in the macaque monkey also project to the mammillary bodies, which may prove to be a reliable feature across species. In the rat, a notable number of CB-positive cells projected to the medial mammillary bodies. Although the calcium-binding proteins assessed in the current study often do not co-localize with one another (e.g., Rogers and Resibois, 1992; Gritti et al., 2003), we could not specifically test whether some of the cells projecting to the mammillary bodies stained for *both* PV and CB. That said, due to the limited overlap between the patterns of expression of these two proteins, it is likely that they represent distinct populations of projection neurons.

One obvious question is: *are all of the neurons in Gudden’s tegmental nuclei that project to the mammillary bodies also PV-positive or CB-positive?* It must first be appreciated that the individual tracer injections into the mammillary bodies could never be complete. Thus, one would expect to find tegmental cells lacking Fluorogold/Fast Blue that nevertheless do project to the mammillary bodies. As such, the degree of PV/CB and retrograde tracer co-localization that we observed will always be less than the true quantity. Therefore, the more informative question is: *did all of the retrogradely labeled cells co-localize with PV/CB*? While many double-labeled cells were found, there were also a considerable number of retrogradely labeled tegmental cells that did not stain for PV or CB. Furthermore, this separation appeared even more prevalent in the dorsal tegmental nucleus. The implication of this finding is that, in the rat, many, but not all, of the tegmental cells that project to the mammillary bodies are PV- or CB-positive, suggesting a neurochemically complex, multifaceted projection (Allen and Hopkins, 1989; Wirtshafter and Stratford, 1993; Gonzalo-Ruiz et al., 1999).

**FIGURE 12:**
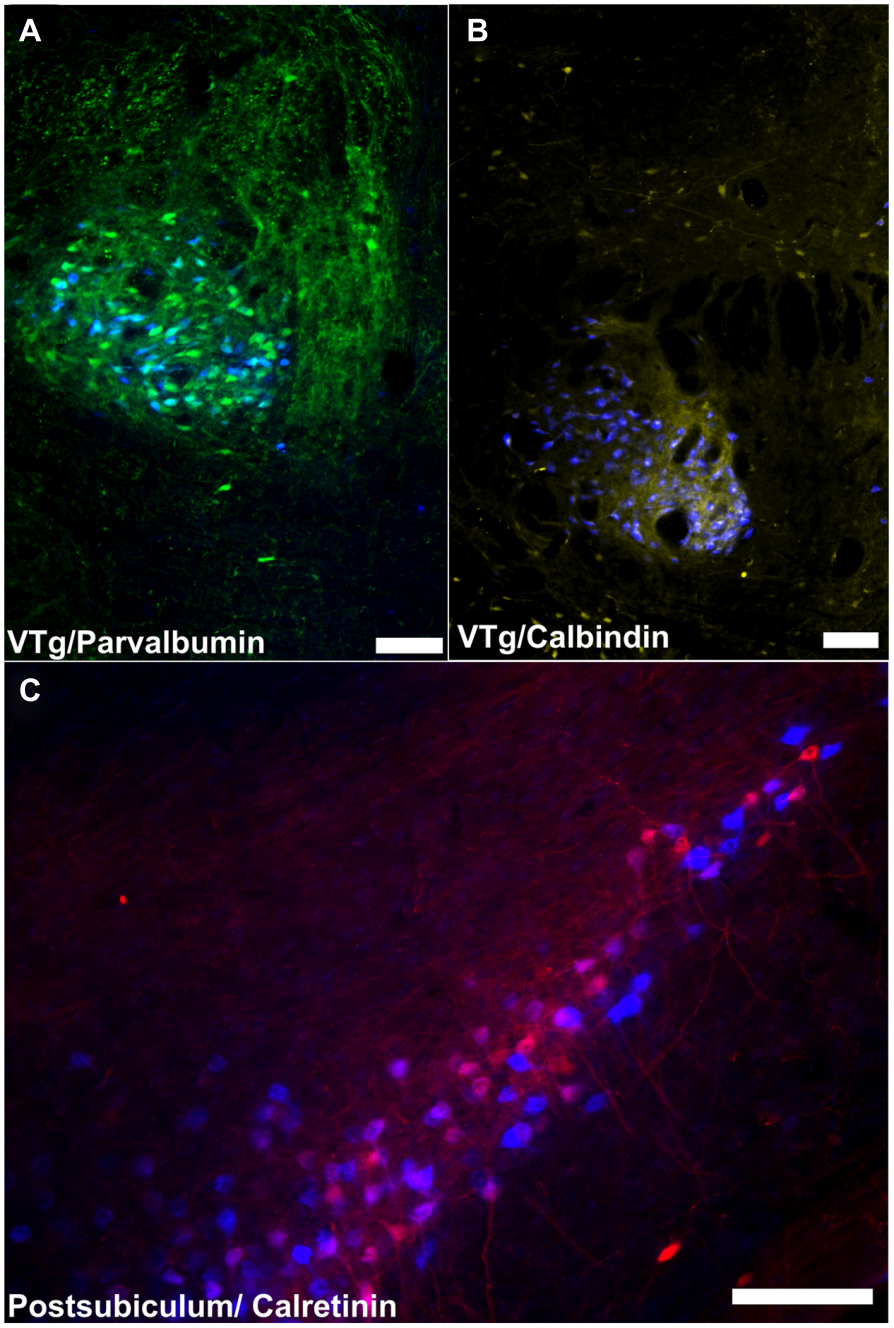
**Summary of calcium binding immunoreactivity in mammillary body inputs. (A)** A considerable proportion of retrogradely labeled cells in the VTg were found to co-localize with PV neuronal immunofluorescence; **(B)** CB immunoreactive neurons in VTg were also found to co-localize with retrogradely labeled Fast Blue cells; **(C)**: Retrogradely labeled Fast Blue neurons were found to co-localize with a dense, localized CR immunoreactive neuronal population in layer III of the postsubiculum. Scale bars: 100 μm.

Although both the dorsal and ventral tegmental nuclei of Gudden contain cells that project to the mammillary bodies and stain for PV, the pattern for these two nuclei appeared quite different. Only VTg showed consistent co-localization between PV and neurons projecting to the mammillary bodies, while the same co-localization was observed far less frequently in DTg and, when present, was restricted to pars ventralis. It is already well established that the ventral and dorsal tegmental nuclei of Gudden exhibit very different functional properties in the rat brain. Neurons in DTg project selectively to the lateral mammillary nucleus and form part of the head-direction system (Hopkins, 2005; Taube, 2007), providing interoceptive cues, e.g., vestibular information (Bassett and Taube, 2001; Bassett et al., 2007), while VTg innervates the medial mammillary nucleus and does not contain head-direction information. Instead, both the medial mammillary nucleus and VTg contain a very high proportion of cells that fire at the frequency of theta (Alonso and Llinas, 1992; Kocsis and Vertes, 1994; Bland et al., 1995; Kirk et al., 1996; Kocsis et al., 2001; van Rijckevorsel et al., 2005). While it was originally proposed that mammillary body theta was driven via descending projections from the septo-hippocampal system (Kirk et al., 1996), it is possible that, in fact, it originates in the midbrain (Bassant and Poindessous-Jazat, 2001; Kocsis et al., 2001; Vertes et al., 2004). Indeed, the present finding, i.e., that VTg neurons projecting to the medial mammillary bodies co-localize with PV (**Figure 12A**), provides some support for this proposal. For instance, it is known that the VTg projection to the mammillary bodies uses GABA (Allen and Hopkins, 1989; Wirtshafter and Stratford, 1993; Gonzalo-Ruiz et al., 1999; Brown and McKenna, 2015). Often found to co-localize, projection neurons that express PV/GABA appear to be of inherent importance for the propagation of rhythmic activity (Borhegyi et al., 2004; Brown and McKenna, 2015; Kim et al., 2015). It is, therefore, possible that the parvalbuminergic projection neurons within the VTg contribute functionally to rhythmical firing in the medial mammillary bodies.

The CB projection from VTg to medial mammillary bodies (**Figure 12B**) is also notable as this seems to be a consistent marker throughout the medial mammillary system (Vann and Aggleton, 2004). Previous studies have reported CB staining in the rodent medial mammillary nucleus and anteroventral/anteromedial thalamic nucleus (e.g., Rogers and Resibois, 1992; Zakowski et al., 2014). Furthermore, the anterior thalamic CB immunoreactivity is thought to originate from the mammillothalamic tract (e.g., Rogers and Resibois, 1992; Zakowski et al., 2014). In contrast, the lateral mammillary system (Vann and Aggleton, 2004), which also contains the DTg and the anterodorsal thalamic nucleus, all show a marked absence of CB immunoreactivity. It has been suggested that CB plays a role in cognition, and memory in particular (Molinari et al., 1996; Emmanuele et al., 2012). CB may, therefore, contribute to these functions within the medial mammillary network.

### Hippocampal Formation

An equivalent investigation into hippocampal formation (subicular and postsubicular) inputs to the mammillary bodies revealed a contrasting pattern of co-localization. While the dorsal subiculum, ventral subiculum and postsubiculum exhibited dense cellular and neuropillar immunoreactivity for PV, immunoreactive cells comprised an almost entirely independent population from those retrogradely labeled neurons that were present.

Calbindin immunoreactivity in the subicular cortices was noticeably sparse (in contrast to the dense immunoreactivity observed in neighboring CA1), with the exception of dense neuropillar immunoreactivity in layer two of the postsubiculum. Consequently, no retrogradely labeled dorsal subicular or postsubicular cells projecting to the mammillary bodies were found to co-localize CB. Noticeably larger numbers of CR immuno-positive cells were present in the subicular cortices, and while none of these cells was found to co-localize with Fast Blue fluorescence in the dorsal subiculum, a substantial number of double-labeled CR immunoreactive cells were found in the postsubiculum (**Figure 12C**). Taken together, this pattern of calcium-binding protein co-localization reinforces the distinction between hippocampal formation inputs to the mammillary inputs and those from Gudden’s tegmental nuclei inputs and further demonstrates the differences between the medial and lateral mammillary inputs.

Recently, studies have highlighted the importance of considering both hippocampal and tegmental inputs when assessing the mammillary bodies’ role in memory. While traditionally, hippocampal inputs have been seen as principally driving mammillary body function, it has been found that selective disconnection of these hippocampal projections to the mammillary bodies has only very modest effects on spatial learning (Vann et al., 2011; Vann, 2013). In contrast, both VTg and DTg lesions produce far more pronounced spatial deficits (Vann, 2009, 2013; Clark et al., 2013; Dwyer et al., 2013). Thus, given the relative impact of damage to the various sources of mammillary body innervation (Vann and Nelson, 2015), the suggestion that Gudden’s tegmental nuclei connections form an inhibitory loop to regulate hippocampal interactions with the mammillary bodies (Allen and Hopkins, 1989; Sutherland and Rodriguez, 1989; Wirtshafter and Stratford, 1993) now seems unlikely.

It is the ascending DTg projections to the lateral mammillary nuclei, not the descending hippocampal projections, which are critical for generating the head-direction signal (Goodridge and Taube, 1997; Bassett et al., 2007); this projection from the postsubiculum does, however, modulate the head-direction signal (Yoder et al., 2015). A similar situation may be true for the medial mammillary system, whereby the VTg is critical for generating medial mammillary theta, perhaps under the modulation of parvalbuminergic VTg input, which, in turn, is modified by descending hippocampal projections.

## Conclusion

By using a combined retrograde neuronal tracing and neurochemical approach, the present study shows that, while a considerable proportion of neurons projecting to the mammillary bodies from the midbrain (VTg) expressed PV or CB, hippocampal inputs to the mammillary bodies did not. Conversely, CR immunoreactivity was present in hippocampal (postsubicular) but not tegmental inputs to the mammillary bodies. Further distinctions were found between the lateral and medial mammillary body inputs. Together, these findings highlight the differences between hippocampal and tegmental inputs to the mammillary bodies, consistent with their functional differences (Vann, 2013; Vann and Nelson, 2015).

## Conflict of Interest Statement

The authors declare that the research was conducted in the absence of any commercial or financial relationships that could be construed as a potential conflict of interest.
